# Fertility recovery of wheat male sterility controlled by *Ms2* using CRISPR/Cas9

**DOI:** 10.1111/pbi.13482

**Published:** 2020-10-07

**Authors:** Huali Tang, Huiyun Liu, Yang Zhou, Hongwei Liu, Lipu Du, Ke Wang, Xingguo Ye

**Affiliations:** ^1^ Institute of Crop Science Chinese Academy of Agricultural Sciences Beijing China; ^2^ College of Agronomy China Agricultural University Beijing China

**Keywords:** Wheat, *Ms2*, germplasm, male‐sterile, CRISPR/Cas9

Wheat (*Triticum aestivum* L.) is one of the most important cereal crops globally. It is essential to develop new wheat varieties for sustainable agricultural production. In the process of wheat breeding, specific germplasm is an essential factor for developing new varieties. Taigu male‐sterile wheat (TMSW) is a special germplasm discovered in China more than 40 years ago (Deng and Gao, 1982). When Taigu male‐sterile wheat is crossed with normal fertile wheat, 50% of plants are male‐sterile and 50% of the plants are fertile in the F_1_ generation. Further study revealed that TMSW contains a dominant male‐sterile gene, *Ms2*, on chromosome 4DS, which controls the complete male sterility of TMSW in heterozygous status (Liu and Deng, 1986). This gene can be easily transferred into different wheat backgrounds for hybridization without artificial emasculation, which is often labour intensive and time consuming, used in traditional breeding programmes.

Further, a dwarf male‐sterile wheat (DMSW) was developed by conventional backcrossing breeding in which *Ms2* was closely linked with a dominant dwarf gene *Rht10* on chromosome 4DS (Liu and Yang, 1991). In the F_1_ generation between DMSW and normal fertile wheat, the short plants are male‐sterile, and the tall plants are fertile. This makes it much easier to distinguish male‐sterile plants from fertile plants simply by the plant height in the breeding population. Using TMSW and DMSW, many new varieties have been developed in wheat through a recurrent selection strategy (Zhai and Liu, 2009). Moreover, *Ms2* has also been transferred to durum wheat and hexaploid triticale (Ji *et al*., 1989; Ni *et al*., 2017).

However, only half of the plants that are fertile in the F_1_ population between TMSW/DMSW and a normal wheat parent can be selected for new varieties and the other half of the plants which are male‐sterile cannot be used for selection. A few years ago, the *Ms2* gene was identified as a reactivated orphan gene due to the insertion of a terminal‐repeat retrotransposon in miniature element in the promoter region of *ms2‐D*, while the other two *Ms2* alleles of *ms2‐A* and *ms2‐B* on chromosomes 4AS and 4BS are pseudogene still in TMSW/DMSW (Ni *et al*., 2017; Xia *et al*., 2017). Recently, an efficient genetic engineering system called clustered regularly interspaced short palindromic repeats (CRISPR) with CRISPR‐associated protein 9 (CRISPR/Cas9) was developed and subsequently widely applied in plants. To date, many wheat genes have been genetically modified using CRISPR/Cas9 (Wang *et al*., 2020).

Here, we used *Agrobacterium*‐mediated CRISPR/Cas9 to edit *Ms2* for restoring the male fertility in sterile wheat lines with excellent agronomic and economic traits for breeding purpose. First, two single guide RNAs (*g7721* and *g9448*) were designed to target exons IV and VII in the *Ms2* open reading frame (ORF) of *Ms2* for CRISPR/Cas9 to increase editing efficiency (Figure 1a). The sgRNAs were synthesized and cloned onto expression vector *pWMBX110‐Cas9* (Figure 1b). Next, immature embryos of the hybrid grains from a cross between DMSW material Jimai22 (*Ms2ms2/Rht10rht10*) and its recurrent parent Jimai22 (*ms2ms2/rht10rht10*) were infected with the *Agrobacterium* carrying the constructed vector (Wang *et al*., 2017). Finally, 133 putative transgenic wheat plants were obtained after co‐cultivation, delay culture and selection culture with phosphinothricin (PPT).

**Figure 1:**
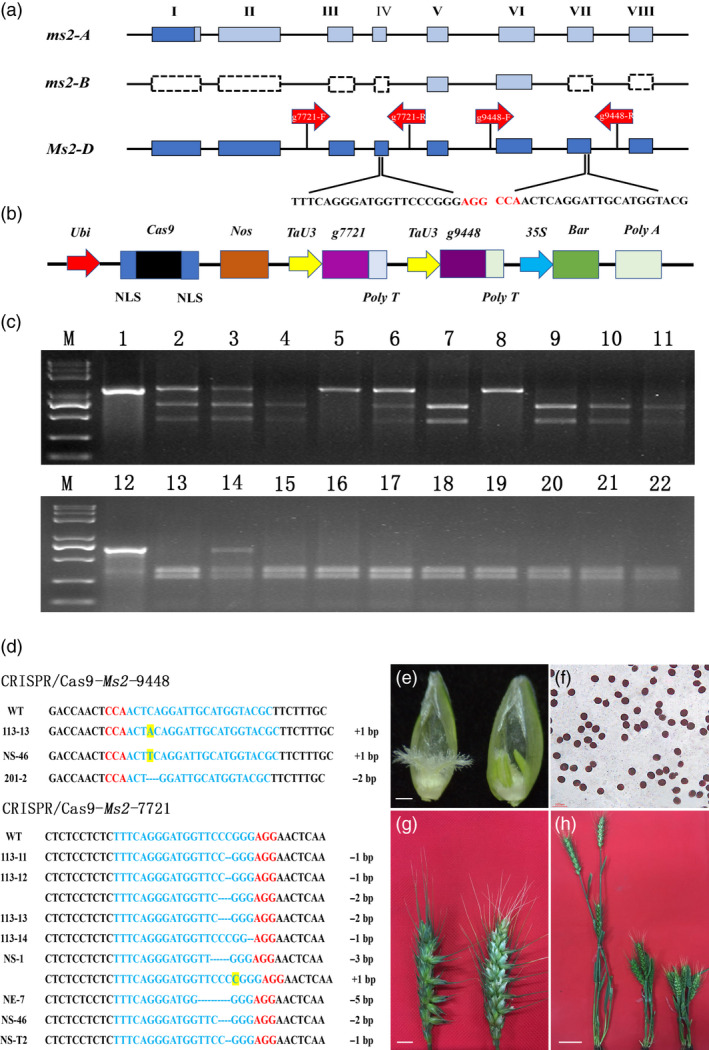
Generation of wheat *Ms2* edited mutant plants using CRISPR/Cas9 and their phenotype and genotype analysis. (a) Structure of three alleles of *Ms2* in DMSW and the targets designed for editing *Ms2‐D*; I to VII: the eight exons of *Ms2*. The dark blue boxes show their ORF; the light blue boxes show the destroyed ORFs; the dashed boxes are the absent exons. The red arrows indicate the detection primers of the two targets; the two base sequences represent the PAM‐guide sequences. The PAM is in red. (b) Linearized structure of vector pWMB110‐Cas9. *Ubi*: ubiquitin promoter; NLS: nuclear localization signal; *Nos*: Nos terminator; *TaU3*: the wheat *U3* promoter; *35S*: *CaMV35S* promoter; *Bar*: phosphinothricin gene. (c) Detection of representative transgenic plants for editing *Ms2‐D* by PCR/RE. M: DNA marker; 1 and 12: the PCR products of transgenic plants without RE; 2‐11 and 13‐22: the digested PCR products of transgenic plants with *Sma*I and *Hpy*188III, respectively. Samples 5 and 8 were homozygous‐edited type, 2, 3, 6 and 14 were heterozygous‐edited type, and 4, 7, 9‐11, 13 and 15‐22 were non‐edited type. (d) Mutant types for target sites 1 and 2 within the sequence of *Ms2‐D* in T_0_ edited plants. The PAM is in red and blue letters indicate sgRNA sequences. The dashed lines represent nucleotide deletions, insertions are shaded in yellow, and black frames represent enzymes sites. The net length for insertions or deletions is presented beside the sequences. (e) The floral organs of DMSW (left) and edited plant (right). Scale bar = 100 μm. (f) Pollen viability stained with triphenyltetrazolium chloride (TTC) of the dwarf edited fertile mutants. Scale bar = 0.1 mm. (g) Appearance of spikes in DMSW (left) and dwarf edited fertile plant (right). Scale bar = 1 cm. (h) Whole plant appearance of tall fertile plant (left), DMSW (middle), and dwarf edited fertile plant (right). Scale bar = 5 cm. [Colour figure can be viewed at wileyonlinelibrary.com]

By polymerase chain reaction (PCR) analysis with primers specific to *Cas9*, all the putative transgenic edited or non‐edited T_0_ plants showing short plant height were detected. Twelve edited plants were screened out further by PCR/restriction enzyme (RE) analysis using *Sma*I and *Hyp*188III and sequencing (Figure 1c,d). Among them, 3 were monoallelic mutants at site *g9448*, of which 2 had a 1‐bp insertion and the other had a 2‐bp deletion in the target sequence; 9 plants were monoallelic mutants at site *g7721*, of which 4 had a 1‐bp deletion, 3 had a 2‐bp deletion, one had a 4‐bp deletion, and one had a 5‐bp deletion; 2 were biallelic heterozygous mutants ((−1/‐2) and (+1/−3)); and 2 had simultaneous mutations at the two sites.

The editing efficiency was 8.27% at site *g7721*, which was significantly higher than that at site *g9448* (2.3%) and the two sites simultaneously (1.5%). The total editing efficiency was 9.0%, in which a mutation occurred in one or two loci. Amplification using *Rht10* specific primers showed that all T_0_ edited plants contained *Rht10*. During spike emergence stage, the edited plants with dwarf phenotype had normal anthers containing viable pollens (Figure 1e,f). During the grain filling period, the edited plants were fertile. As expected, the fertile dwarf mutants showed similar plant height to DMSW (Figure 1g,h).

To detect the mutations inheritance, we planted the edited mutants and analysed T_1_ plants using PCR/RE assay for the target sites of *Ms2* and PCR analysis for *Rht10*. We found that the mutations on the targeted sites were continuously identified and all mutant plants contained *Rht10*. In addition, the edited T_1_ plants showed pistils and normal anthers while the DMSW plants had pistils but no anthers. The edited T_1_ plants with similar height to DMSW were fertile. Additionally, about one‐fourth of the T_1_ edited plants with genotype *ms2ms2/rht10rht10* were tall due to the segregation of *Rht10*. The T_1_ edited plants with genotype *ms2^e^ms2^e^/Rht10Rht10* were not segregated.

All the above results indicated that the fertility of DMSW was completely recovered through editing *Ms2* using CRISPR/Cas9. Our results also supported the precise cloning of *Ms2* in DMSW (Ni *et al*., 2017; Xia *et al*., 2017). But, the editing efficiency in this study is lower than that in our previous report on the editing of wheat *TaMTL* (Liu *et al*., 2020). Firstly, DMSW can be only maintained by crossing it with a fertile wheat, and thus the hybrid grains consist of two genotypes *Ms2ms2/Rht10rht10* and *ms2ms2/rht10rht10* with the same ratio of 50%; but only the grains with *Ms2ms2/Rht10rht10* are available materials for editing *Ms2* and the grains with *ms2ms2/rht10rht10* are not the targeted materials but transformed at the same time because they cannot be distinguished. Secondly, the genotype *Ms2ms2/Rht10rht10* controlling the dwarf male‐sterile trait is heterozygous at this locus, and the original *ms2* being a pseudogene, the allele of *Ms2*, might be also edited once it was targeted by the sgRNAs for knocking‐out, which did not affect the fertile phenotype. Thereby, the editing efficiency for *Ms2* using DMSW is only one‐fourth of that for other target genes using normal wheat varieties.

In summary, *Ms2* was successfully edited and the fertility of DMSW was completely recovered by CRISPR/Cas9. The editing efficiency was 9.0%. The fertile mutants obtained from the male‐sterile lines with excellent agronomic traits, biotic and abiotic resistance and better quality feature can be selected for developing new varieties. This strategy can also be extended to restore the fertility of male‐sterile durum wheat and hexaploid triticale lines containing *Ms2* with good performance for the same purposes.

## Conflict of interest

No conflict of interest declared.

## Author contributions

X.Y. and K.W conceived the project. H.L. constructed plasmid. K.W. and L.D. performed transformation. H.T. and H.L. detected edited plants and performed data analysis. H.T., Y.Z., H.L. and X.Y. made crossing and investigated phenotype. H.T. and X.Y wrote and revised the manuscript.
